# Clinical Signs of Kawasaki Disease from the Perspective of Epithelial-to-Mesenchymal Transition Recruiting Erythrocytes: A Literature Review

**DOI:** 10.31083/j.rcm2404109

**Published:** 2023-04-17

**Authors:** Jin-Hee Oh, Soyun Cho, Jin A Choi

**Affiliations:** ^1^Department of Pediatrics, St.Vincent's Hospital, College of Medicine, The Catholic University of Korea, 16247 Seoul, Republic of Korea; ^2^Department of Dermatology, Boramae Medical Center, College of Medicine, Seoul National University, 07061 Seoul, Republic of Korea; ^3^Department of Ophthalmology & Laboratory of Visual Science, St.Vincent’s Hospital, College of Medicine, The Catholic University of Korea, 16247 Seoul, Republic of Korea

**Keywords:** mucocutaneous lymph node syndrome, epithelial-to-mesenchymal transition, innate immunity

## Abstract

Kawasaki disease (KD) is a systemic vasculitis affecting children younger than 5 years of age. Early period in life is marked by rapid somatic growth with cell proliferation and immaturity of the immunity with dominant innate immune system. Coronary complications in KD are the most common acquired heart disease in children, yet the diagnosis of KD still depends on the clinical diagnostic criteria. Glossy red lips and conjunctival injection are characteristic signs enabling pediatricians to make the initial diagnosis of KD; however, little is known why these are so characteristic. The diagnostic criteria of KD seem to be scattered in seemingly irrelevant body systems such as the eyes, lips, skin, and heart. KD is classified as a connective tissue disease. Recently, red blood cells (RBCs) have emerged as important modulators in innate immune response. RBCs are reported to participate in extracellular matrix remodeling and upregulating matrix metalloproteinase (MMP) expression in dermal fibroblasts. Also, fibroblast growth factors and microRNAs associated with fibrosis are drawing attention in KD. The cardinal signs of KD appear at the border of muco-cutaneous junction. Head and neck regions are abundant in tissues undergoing epithelial-to-mesenchymal transition (EMT). Interstitial carditis and valve insufficiency as well as coronary arterial lesions may complicate KD, and these lesions present in tissues that originated from epicardial progenitor cells by EMT. Having reviewed the recent research on KD, we presume that the signs of KD present at borders between keratinized and non-keratinized stratified squamous epithelium where the EMT is still ongoing for the rapid somatic growth where RBCs are recruited as an innate immune response and to prevent excessive fibrosis in mucosa. KD presents scarcely in adults with somatic growth and immune maturation completed. In this review, we attempted to explain the reasons for the clinical manifestations of KD and to search for a link among the diagnostic clues in the perspective of EMT during the somatic growth and immune system maturation in children with KD.

## 1. Introduction

Kawasaki disease (KD) is a mucocutaneous lymph node syndrome [1], typically affecting children younger than 5 years of age, and histologically is a systemic vasculitis affecting medium-sized vessels [2]. A number of papers have updated the knowledge on cytokines and genes in KD [3], and many papers are trying to explain the pathophysiology of KD by correlating unexplained clinical symptoms and laboratory findings [4]. As an etiology, infection seems to trigger the onset of symptoms in a genetically susceptible group of children and the efforts to find the etiology of KD are still ongoing, and many studies related to the causative virus have been reported [5]. During the recent COVID-19 pandemic, it has been reported that the annual incidence of KD has significantly decreased in Korea [6].

According to the Japanese report of annual frequency of cardiac sequelae of KD in the years of 2015–2016, 1.30% of patients had coronary dilatation, 0.64% had aneurysm, 0.13% had giant aneurysm and 0.023% had myocardial infarction. Pan-vasculitis in the coronary artery occurs around the 10th day of onset of KD, and the dilated coronary arterial lesion due to coronary vasculitis forms around the 12th day of disease [7]. Coronary complications of KD are emerging as the most common acquired heart disease in children, yet the diagnosis of KD still depends on the clinical diagnostic criteria. Glossy red lips and conjunctival injection are so characteristic that they enable pediatricians to make the initial diagnosis of KD, and these features distinguish KD from other necrotizing systemic vasculitis, such as Henoch-Schönlein purpura. However, little is known why these findings look so characteristic compared to other vasculitides or other infectious diseases. The diagnostic criteria of KD seem to be scattered in mutually irrelevant body systems such as the eyes, lips, cervical lymph nodes, finger and toe tips, Bacille Calmette-Guérin (BCG) injection site and heart, most predominant on the head and neck. KD is now classified as a connective tissue disease. Clinical manifestations and diagnostic imaging findings suggest vasculitis leading to edema of connective tissue. Recently, red blood cells (RBCs) are emerging as important modulators in the innate immune response [8]. CD71+ erythroid can affect the different functional properties on monocytes or dendritic cells [9, 10]. Also, fibroblast growth factors are gaining attention in KD as well as microRNAs related to the fibrosis identified in KD [11]. The acquired immune system and skin keratinization are not yet completed in young children when the proportion of premature RBCs is high as well. Compared to adulthood, this early period in life is marked by rapid somatic growth with cell proliferation from the intrauterine period until the completion of growth in the adult age. During the infantile period, body weight of a 12-month-old infant is triple that of birth weight. Rapid somatic growth is observed in cornea and digits. Bergmann *et al*. [12] reported the dynamics of human heart cell generation showing that the numbers of both endothelial and mesenchymal cells increase substantially from birth to early adulthood, whereas the full complement of cardiomyocytes is established perinatally and remains stable over lifespan. Among these transitions, there are differences in the velocity of proliferative cell growth in the integumentary system, and lymphatic and immunologic system, which seems to be related to the phenotype of KD according to patients’ age.

We might have underestimated the cues of typical vivid red color of vermillion and conjunctival injection which appear abruptly and resolve without a long-term complication. Therefore, conjunctival injection and red lips recruiting RBCs can be a cardinal clue of KD prevalent in young children whose innate immunity is majorly orchestrating to cope with the etiology of KD. The cardinal signs of KD appear at the border of muco-cutaneous junction. Head and neck area is abundant in tissues that undergo epithelial-to-mesenchymal transition (EMT) while neural crest cells migrate into their fate to head and neck. Recent study demonstrated that human keratin-14+ keratinocytes can form neural crest cells by reprogramming postnatal human epidermal keratinocytes toward functional neural crest fates [13]. Subungual and perianal desquamation in KD starts from the border between the epithelium and mucosa at the end of nail bed and perianal area.

Heartwise, interstitial carditis and valve insufficiency as well as coronary arterial lesions in KD share the common features that the involved tissues have originated from the epicardial progenitor cells by EMT. The cardiac lesions in KD are predominant in the interstitium, compared to myocardium, and that mitral regurgitation and pericardial effusion are predominant in imaging studies during the acute phase of KD. Ultimately, the coronary aneurysms start from the intima of coronary artery in KD [14]. This review attempts to explain the reason for the clinical features of KD, searching for a link among the diagnostic clues of KD with a developmental point of view focusing on the innate immunity with a role of RBCs and EMT in children with KD.

## 2. Methods

The purpose of this paper was to find a common link of clinical symptoms presenting in different organs in KD from the EMT point of view. We searched PubMed, MEDLINE, and EMBASE for relevant clinical and basic experimental studies published in English since 1990. We tried to find a clue of EMT to each symptom of KD, and we searched for data using keywords: “Kawasaki disease”, “epithelial to mesenchymal transition”, “conjunctiva”, “cornea”, “lips”, “BCG”, “cardiac development”, “epicardial cell”, and then, “mucosa”, “fibrosis”, “wound healing”, “erythrocyte”, “red cell distribution width”, etc. for similar and/or combinations of those words. Among many papers, after removing duplicates, eighty-three papers whose contents could suggest EMT and body tissue growth in children were mainly selected.

## 3. Results

### 3.1 Emerging Evidence of the Role of Fibroblasts and EMT in KD

EMT has been a hot topic in relation to the pathogenesis of various diseases. The major roles of EMT are mentioned in embryonic development, somatic growth and wound healing, tissue regeneration, and organ fibrosis [15]. When wound occurs, the skin and mucosa go through inflammation, proliferation and remodeling. Granulation tissue forms in inflammatory environment and progresses to proliferation stage, where keratinocytes constructing barrier and fibroblasts secreting extracellular matrix and remodeling granulation tissue migrate to the wound bed [16]. Dermal fibroblasts are cells generating connective tissue allowing the skin to recover from injury. Like corneal fibroblasts, dermal fibroblast proliferation is stimulated by fibroblast growth factor (FGF) [17]. Dermal fibroblasts are derived from mesenchymal stem cells and can give rise to myofibroblasts with smooth muscle characteristics. Peng *et al*. [18] reported increased levels of circulating fibroblast growth factor (FGF)-21 in children with KD. This report showed the potential role of serum FGF-21 in KD; its levels were significantly increased during the acute phase of KD and higher in KD with coronary arterial complication, while serum levels of RBC and albumin were decreased in the KD group with coronary complication.

Also, elevated FGF and ferritin are known for the occurrence of cardiac complication in children with KD [19]. Recent study on the microRNA (miRNA) in KD showed that the MIT-24-3p plays a critical role in KD progression and the authors mentioned that these miRNAs were significantly involved in the transforming growth factor-β (TGF-β), epithelial-mesenchymal transition, and cell apoptosis signaling pathways [11]. The miR-24 regulates cardiac fibrosis by modulating the TGF-β pathway [20].

Stratified squamous epithelium in the body is classified as two parts; keratinized and non-keratinized. Examples of keratinized epithelium are epidermis and cornea, while non-keratinized epithelium includes oral cavity, conjunctiva, upper one-third esophagus, rectum, and female external genitalia. The transition zone of vermillion is at the border of the lips making the ‘red-line’ of lips. Vivid red lips and conjunctival injection as typical cardinal symptoms and signs of KD appear at the border of muco-cutaneous junction, in other words, between the non-keratinized epithelium (oral mucosa and conjunctiva) and the keratinized stratified epithelium (skin and cornea) where dermal and corneal fibroblasts are found, respectively.

During the embryonic development, coronary smooth muscle originates from the proepicardial cells, while cardiac muscle fibers and most smooth muscles are derived from visceral mesoderm. EMT of epicardial cells leads to the formation of epicardially derived cells that migrate into the ventricular myocardial walls where they differentiate into interstitial fibroblasts and coronary smooth muscle cells. In addition, the epicardially derived cells contribute to the leaflets of the atrioventricular valves that are driven from the lateral atrioventricular cushion [21, 22, 23].

In neonates or young infants, the keratinization is incomplete, in other words, the skin is weakly protective. Recent study showed differences in skin between human infants and adults in epidermal development, e.g., keratinocyte differentiation, keratinization and filament cytoskeleton organization, which involve immune function, including antigen presentation [24]. Hence, during the infantile period additional protective measures might be required.

### 3.2 Emerging Evidence of RBC Acting as Modulators of Innate Immunity

In inflammatory process, it is known that iron metabolism in hematopoiesis changes and the maturation of RBC is affected. The important role of innate immunity in KD is suggested in the mouse model [25]. Also, recent interesting study offered a perspective of human RBCs emerging as important modulators of the innate immunity and discussed their activities in sepsis [8]. Hemoglobin (Hb) and heme are facets of innate immunity, generating antimicrobial reactive oxygen species (ROS) to defend against invading hemolytic microbes as well as promoting inflammatory and autoimmune response.

During the evolution of RBCs in the body, every RBC has a nucleus which is enucleated later. The enucleated reticulocytes are then released into blood stream to complete the maturation process until the RBCs are cleared by macrophages in the spleen and liver after about 120 days of lifespan [26]. These erythrocytes are able to interact with inflammatory molecules and pathogens, regulating and modulating immune responses. Nucleated RBCs appear in the peripheral system and the level of ferritin goes up at serious inflammatory status. Red cell distribution width (RDW) is an indicator of volume and size of RBC. RDW reference intervals for neonates are higher than for older children with the upper reference limit of term RDW being 20%, and higher (up to 23%) in preterm neonates [27]. Nucleated RBCs are typically observed in the peripheral blood stream of fetuses and neonates [28] and known for a direct response to mediators in inflammation in newborns with early-onset neonatal sepsis [29]. Immature nucleated RBCs express CD71 surface marker and these immature erythroid precursors have also been reported in healthy adult peripheral blood [30]. Interestingly, it is known that the immunosuppressive CD71+ erythroid cells compromise neonatal host defense against infection [9]. Recently, papers have been published on the relations of RDW and vascular aging biomarkers and endothelial progenitor cells related to the cardiovascular disease [31, 32]. Studies suggesting the association of elevated level of RDW with disease activity in other systemic vasculitis such as Henoch-Schönlein purpura have been increasing [33, 34, 35, 36]. Papers indicating the importance of RDW as a predictor of coronary arterial lesions in KD are also increasing [37, 38, 39, 40]. Pediatric patients with KD, septic shock, macrophage activation syndrome and many other conditions requiring differential diagnosis show anemia in overwhelming conditions [41]. Also, combination of Hb for age Z score and plasma hepcidin was suggested as a predictor for KD [42]. Ferritin in pediatric critical illness is drawing attention [43]. Hyperferritinemia is highly specific and sensitive for detecting macrophage activation syndrome in children with KD [44]. RBCs are also known as dynamic reservoirs of cytokines [45].

Fibroblasts are major cellular component of healing wounds and paracrine signals may influence the collagen/matrix metalloproteinase (MMP) balance in resident fibroblasts. Proteins expressed in RBCs in infants are known to be different from those in adults. Kilani *et al*. [46] showed that, among RBC proteins, isoforms are expressed differently according to the ages and the levels of some of these proteins are higher in RBCs of newborn babies compared to those of adults. They found that circulating monocytes stimulated to be transformed into “keratinocyte-like cells” could promote an anti-fibrogenic commitment of dermal fibroblast via exosomal 14-3-3 proteins. And RBCs containing 14-3-3 proteins significantly increased the expression of MMP-1 in dermal fibroblasts concluding the RBC lysates might play an important role in the regulation of extracellular matrix [46].

Taken together, clinical manifestations in KD may present as a consequence of recruiting RBCs in the non-keratinized stratified epithelium in KD. In this regard, we will try to explain the clinical features of KD in every body system one by one in the following sections.

### 3.3 Glossy Red Lips in KD

**Fig. 1.**
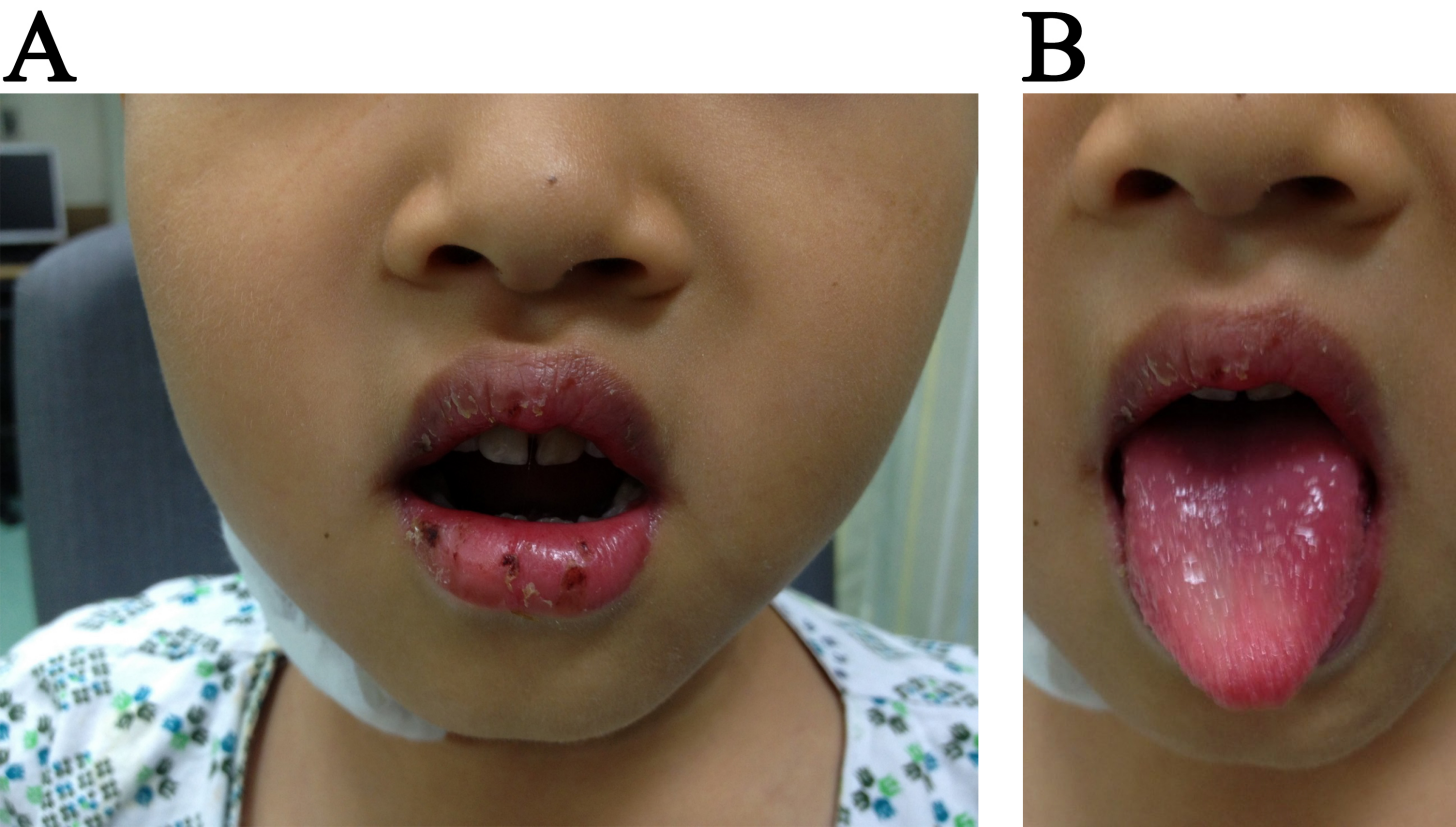
**Characteristic glossy red lips with vertical bloody fissures (A) and strawberry tongue (B) in a 5-year-old patient with KD**. KD, Kawasaki disease.

KD is prevalent in children younger than 5 years of age when the innate immunity dominates in survival strategies and the acquired immunity is still developing. Many KD patients show the red lips and strawberry tongue, which are manifested by the recruitment RBCs, a hallmark of diagnosis of KD (Fig. 1A,B). Then, why are the red lips in KD so glossy, compared to red lips in other febrile illnesses or in other vasculitides? Glossy red lips in KD might be due to the increased filling volume of vermillion by recruited RBCs rendering the natural crease of lips to be stretched out, and the reflection of recruited RBCs against connective tissue cells causes the lips to appear glossy. The stiffness of lip mucosa in KD on the non-keratinized stratified squamous epithelium leads to a tear forming typical vertical fissures by traction force horizontally toward mouth angles when the baby cries. For ethical issues, it is not feasible to obtain a biopsy specimen from red lips during the acute phase of KD. Spectral reflectance curve with spectrophotometer quantifies Hb in the lips and skin. However, any contact of probe of spectrophotometer on the lip surface may shift and disperse the RBCs from the contacted spot, so it is not easy to get accurate data in color measurement of the vermillion.

The characteristic of normal oral mucosa exposed to mechanical abrasion and tension is that it heals much faster with less scarring than the skin [47]. The reason seems that oral mucosa fibroblasts and dermal fibroblasts have different cell behaviors to growth factors. When oral mucosa fibroblasts have a higher proliferation rate, they have a lower shrinkage capacity and synthesize more collagen when exposed to TGF-β1 [48].

Infant’s RBCs are known to be different from those of adults in that, they act as innate immune modulator and express different proteins. During the baby’s somatic growth, EMT is underway at the border of keratinized epithelium bordering the non-keratinized vermillion and tongue toward the RBCs with high RDW recruited as an innate immune response. Also, as oral mucosa should be healed rapidly with less scar formation under pathogenic environment, RBCs containing antifibrogenic factors may be recruited to the non-keratinized oral mucosa in KD to regulate the EMT by mucosal fibroblasts as mucosa needs fast healing process with less scarring.

Connective tissue forming scaffold of the structure that undergoes EMT looks highly glossy. Tendons, pericardium, pleura, intima of vessels, and scar tissues are typical examples of connective tissue, and all of them look highly glossy in the body. The recruited RBCs in the connective tissue may appear highly glossy red. As enucleated erythrocytes cannot transcript genes or synthesize proteins, they express a large number of receptors interacting with exogenous agents in the blood, scavenging or sequestrating the circulating molecules in innate immunity. Hb stimulates macrophage tumor necrosis factor (TNF) production and triggers the release of pro-inflammatory cytokines [49]. In clinical course of KD, red lips of young patients are a harbinger of ensuing systemic inflammation with cytokine surges.

### 3.4 Skin Rash and Erythematous Change in Inoculation Site of BCG

The characteristics in skin lesions in KD are well described in other papers [50]. Skin biopsies performed on exanthema in KD cases showed extensive edema and infiltration of mononuclear cells including T lymphocytes and macrophages in the papillary dermis [51].

The erythema in BCG site is one of the hallmarks of KD in young children. However, it is little known why erythematous lesion appears at BCG inoculation site. The incidence of erythema in BCG sites depends on the patient’s age, and Kim reported that BCG erythema developed more frequently in infants rather than grown children, appearing in 73% of infants (≤5 months), 34% among 6 mo–4 yrs of age, and only 3% of older children (≥5 yrs) with KD [52]. Amongst BCG-vaccinated children, having a BCG scar is known for better survival compared with not having a scar and the effect on survival was particularly strong when BCG had been administered in the neonatal period. Recently, factors initiating EMT are explained in acute and mild trauma, that wounded epithelial cells differentiate into fibroblast-like cells to produce tissues and organs, which is a reparative biological process [53].

The biopsy specimen at the BCG injection site of two 5-month-old infants after the treatment of KD showed infiltration of inflammatory cells without granuloma or leukocytoclastic vasculitis in the infant who received BCG 1 week prior to the initiation of KD, and infiltration of inflammatory cell and epithelioid granuloma without caseous necrosis in the infant who received BCG 6 weeks prior to the initiation of KD [54]. Also, Araki *et al*. [55] reported that BCG site erythema was observed in patients with KD who developed the onset of KD symptoms from 31 to 806 days after BCG vaccination with a hazard ratio of 0.995, 95% confidence interval = 0.993–0.997. In many countries including South Korea, BCG vaccination is adopted as a nation-wide mandatory program for infants. Among the vaccines for infants, BCG is the one inoculated into the dermis while most other vaccinations into subcutaneous tissue. Infants with KD show the typical erythema at BCG scars. This phenomenon declines with the growth of children when prominent firm fibrotic scar has formed in the BCG inoculation site. As with conjunctiva and lips, recruited RBCs may be recruited to the papillary dermis as a modulation of innate immunity, and play a role in balancing collagen and MMP by fibroblasts in rapidly growing infants, rendering erythema at the inoculation site of BCG.

Also, finger and toe tip erythematous edema is observed in KD, and it is at the border of skin epithelium of digits and subungual area where the non-keratinized epithelial surface is covered with extremely thickened stratum corneum called nails where EMT is active. Periungual full thickness desquamation during the convalescent stage of KD is one of the hallmarks of KD. A study reported the subungual desquamation to occur in 68% [56]. The cardinal symptoms and signs of KD appear at the border of muco-cutaneous junction; thick subungual desquamation appears precisely at the area where epithelium and mucosal border toward the RBCs are recruited to make the finger and toe tips erythematous and edematous, and finally, the disassembly of adhesion junction starts at the boundary. Perianal desquamation may also be explained in the same way.

### 3.5 Conjunctival Injection in KD

**Fig. 2.**
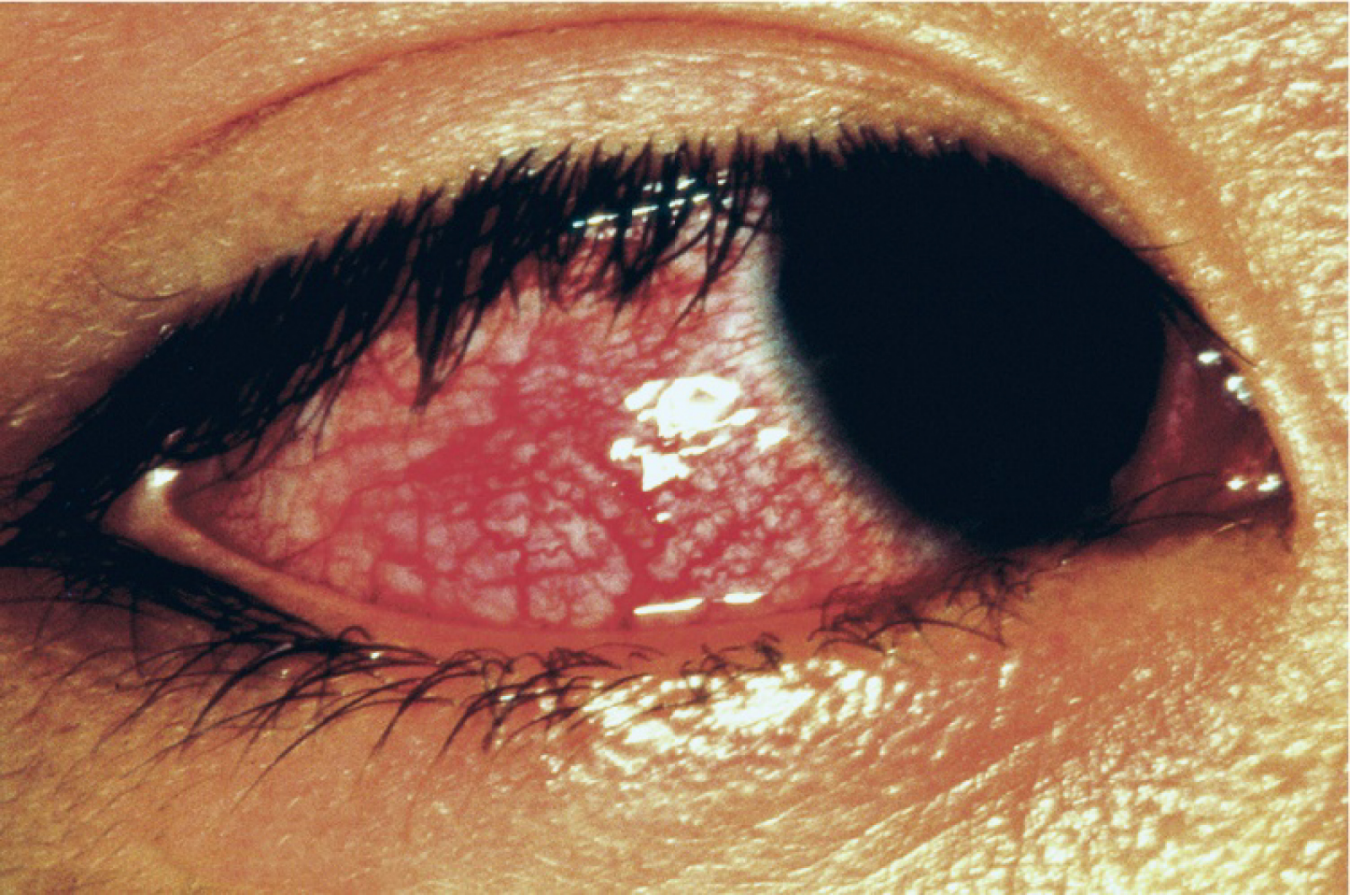
**Bilateral bulbar conjunctival injection without exudate typically observed in patient with KD**. Reproduced with permission from [57]. KD, Kawasaki disease.

During the clinical course of KD, conjunctival injection presented within first 2 days after the onset of fever (Fig. 2) [57]. Characteristically, red conjunctiva in KD is promptly recognized at a glance through the naturally transparent conjunctiva contrasted to the underlying white sclera.

During development, corneal dimensions in the term “newborn eye” are closer to the adult dimensions but become thicker during the first several months of life from enlargement of the collagen fibrils and once mature, the collagen fibers no longer thicken during subsequent aging [58]. Cornea is made of multilayered stratified squamous epithelium, corneal endothelium and the corneal keratocytes, specialized corneal fibroblasts that reside in the stroma. Keratocytes are developmentally derived from neural crest cells which underwent EMT. Human limbal mesenchymal cells support the growth of human corneal epithelial progenitor cells [59].

Conjunctiva is a mucous membrane out-skirting cornea. It is a morpho-functional unit supported by gatekeepers such as antigen-presenting cells and T-lymphocytes of the eye. Conjunctiva clears pathogens and allergens. Conjunctiva is originally derived from the ectoderm and underwent EMT during the embryonic development. The conjunctival tissue is highly prone to undergo EMT upon injury. Under pathologic conditions, the process of EMT reappears in conjunctiva rendering epithelia to acquire features of mesenchymal cells, characterized by loss of epithelial features including keratin expression, apico-basal polarity, disassembly of adhesion junctions, etc. [60]. A recent study elucidated the molecular mechanism by which conjunctival epithelia, which arise from ectodermal cell, dictate cell fate and ability of EMT [60]. The authors showed that corneal and conjunctival epithelia arise from a common ancestral ectodermal cell, then diverge into distinct lineages.

Corneal macrophages are classified as two types, C-C chemokine receptor (CCR) 2– and CCR2+. The former shows local proliferative capacity, and depletion of CCR2– macrophages increases inflammation of the injured cornea; the latter acts conversely [61]. The study showed two unique macrophages in the cornea participating in corneal wound healing by balancing inflammatory response. Conjunctival injection in KD is a non-exudative transient bilateral conjunctival injection typically involving the bulbar conjunctiva in contrast to the palpebral conjunctiva affected in another viral conjunctivitis. Bulbar conjunctiva is a continuum of non-keratinized squamous epithelia whereas the palpebral conjunctiva is made up of columnar epithelium, not squamous epithelium.

Naturally, conjunctival tissue is highly prone to undergo EMT upon injury. The differential ophthalmic diseases showing similar features to KD include Stevens-Johnson syndrome (SJS). However, severe conjunctival fibrosis occurs in SJS. Given the fact that the conjunctival tissue shows little or no inflammation and that it improves spontaneously without any sequela, red conjunctivae in KD is due to congestion by recruited RBCs rather than true vasculitis. There are no symptoms suggesting conjunctivitis, such as chemosis, follicles, papillae or membrane formation in KD. Despite the vulnerability of conjunctival epithelium to EMT, it is curable without fibrosis and other sequalae in KD. As conjunctiva should be healed rapidly with less scar formation in pathologic conditions in very young patients, RBCs are recruited to the conjunctiva, non-keratinized epithelium, to regulate the EMT by fibroblasts possibly causing red conjunctivae in KD.

### 3.6 Acute Non-Purulent Cervical Lymphadenopathy in KD

Lymph node (LN) is encapsulated by a capsule composed of dense irregular connective tissue. Lymphatic drainage of conjunctiva and lips is known for majorly draining into superior deep cervical LNs [62]. Cervical LNs are predominantly affected in KD and other LNs in the axillary or inguinal area are less affected. Head and neck lymphatic tissue are known for undergoing EMT during the migration of neural crest cells from the pharyngeal arches to their fate in the head and neck. Enlargement of cervical LNs is common in KD, whereas enlargement of the tonsils is less common. The difference is that tonsils belong to the extranodal lymphoid tissue and do not have a capsule.

**Fig. 3.**
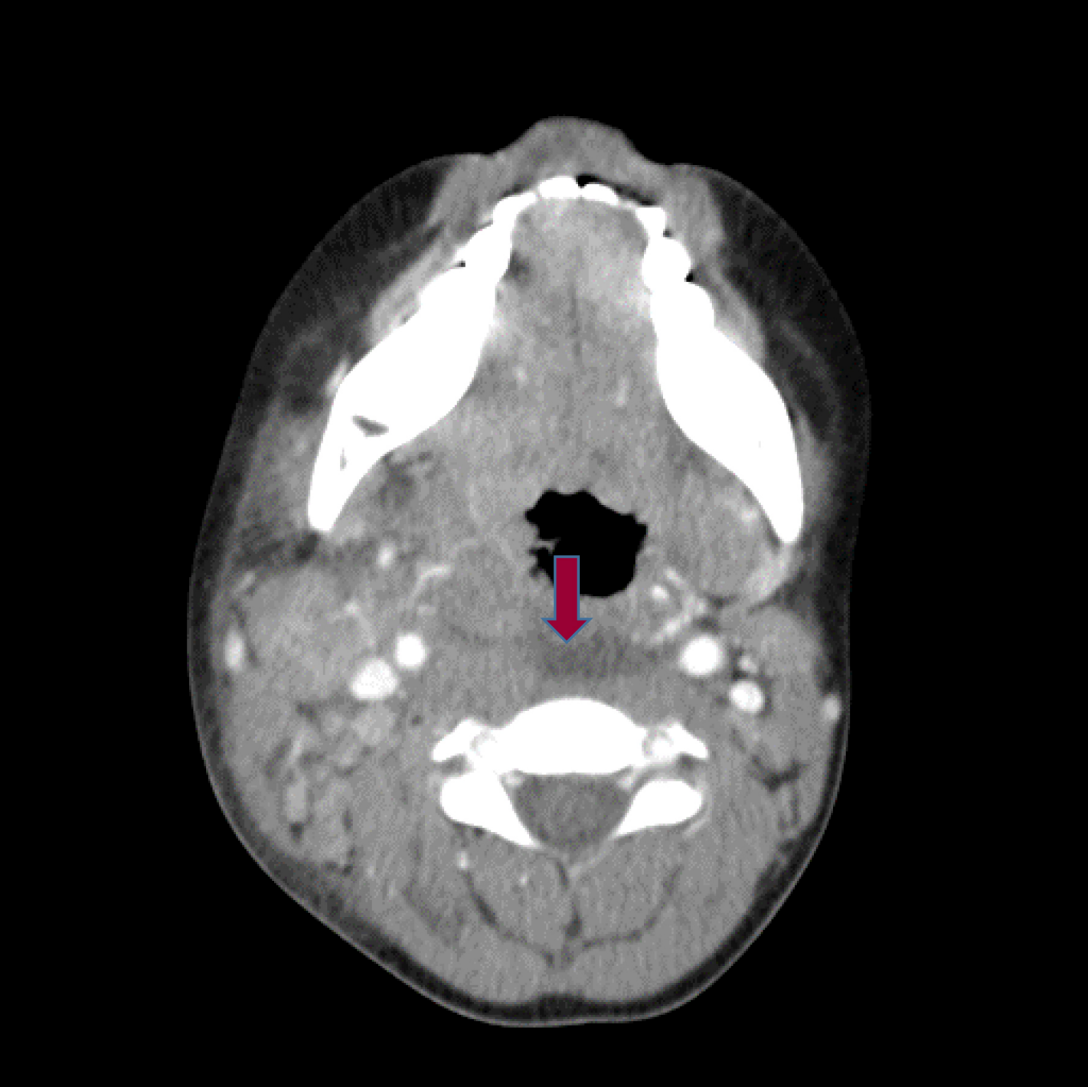
**Non-suppurative cervical lymphadenopathy with retropharyngeal phlegmon (arrow) in a 5-year-old patient with KD**. KD, Kawasaki disease.

Among the diagnostic criteria, the presentation of lymphadenopathy is less observed in very young infants and more frequently observed in older children. Some older children show LN enlargement first prior to the presentation of other diagnostic features, and this phenotype of KD is often called node-first KD. Some patients with node-first KD demonstrate transient erythema on the skin over the affected cervical LNs. Children with node-first KD tended to be older (4 vs 2 years) and had more days with fever and higher CRP levels [63]. An explanation for this may lie in the fact that the lymphatic system develops later in children [64]. Imaging study with computed tomography (CT) on involved cervical LN shows peritonsillar hypodense area [65]. Some patients show retropharyngeal phlegmon (Fig. 3). Papers comparing the lymph-node-first KD with bacterial cervical adenitis and typical KD showed that patients with node-first KD group were older compared to other groups and showed multiple solid nodes with comparable rates of retropharyngeal edema [63]. Biopsy specimen of cervical lymph nodes in acute phase of KD showed non-specific findings, with high degree of non-purulent inflammation in the LN capsule and surrounding connective tissue featuring mainly monocytes or macrophages [66]. KD is categorized as connective tissue disease, and thus cervical lymphadenopathy is characteristically non-suppurative.

### 3.7 Cardiac Lesions in KD 

Cardiac cell proliferation from the perspective of EMT may be a clue to the predilection for cardiac complications in the connective tissue of the heart and coronary arteries. Bergmann *et al*. [12] revealed a high turnover rate of endothelial cells throughout life and more limited renewal of mesenchymal cells in adulthood. They also showed that the cardiomyocyte numbers are constant throughout the human lifespan, with a low turnover rate. Cardiomyocyte exchange is highest in early childhood and decreases gradually to <1% per year in adulthood. However, endothelial and mesenchymal cells are exchanged at a high rate, and their numbers increase into adulthood.

**Fig. 4.**
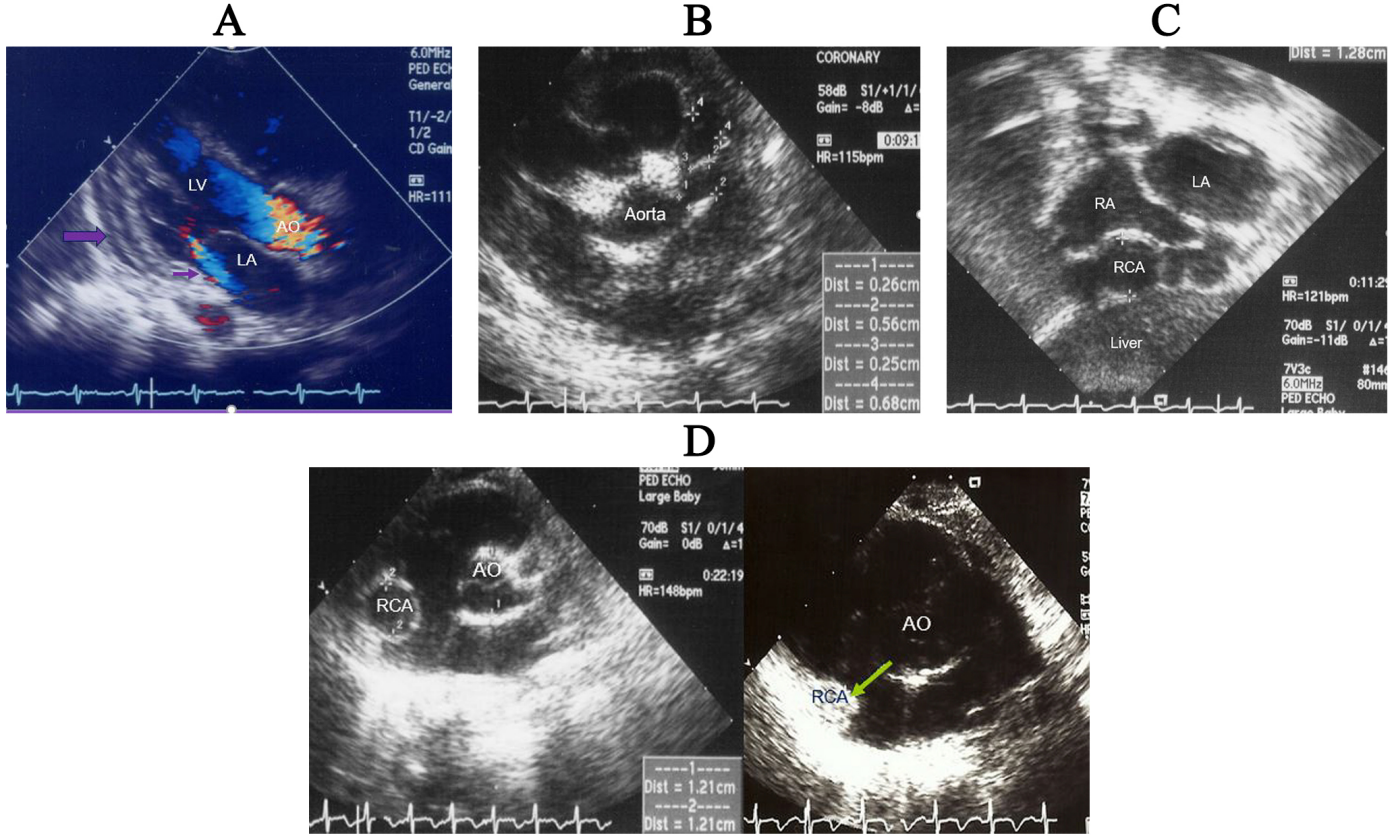
**Echocardiographic images of a 3-month-old infant with KD**. Parasternal long axis view with color Doppler showing mitral valve regurgitation (small arrow) and pericardial effusion (Large arrow) (A), parasternal short axis view showing fusiform coronary aneurysms of the left coronary arteries (B), modified subcostal view showing giant coronary aneurysm of the right coronary artery (C) and parasternal short axis view showing right coronary giant aneurysm of which inner diameter is as large as aorta (left) and complete intraluminal occlusion (arrow) 1 year later (right) (D). (B,C) are reproduced with permission from [68]. KD, Kawasaki disease; LV, left ventricle; LA, left atrium; AO, aorta; RA, right atrium; RCA, right coronary artery.

The pathogenesis of KD arteritis is characterized by granulomatous inflammation with accumulation of monocytes/macrophages [67]. Clinically, frequent cardiac manifestations in KD include myocarditis, cardiac valvulitis, pericardial effusion (Fig. 4A), and coronary arterial lesions including aneurysmal formation (Fig. 4B,C) [68], subsequent stenosis or complete occlusion of the lumen (Fig. 4D) of coronary arteries. All of these cardiac presentations have in common that involved cells have undergone EMT during cardiac development. The expanded role of epicardium and epicardial-derived cells in cardiac development and disease has been reviewed [69].

What distinguishes carditis of KD from the carditis caused by other viral infections is that it is preferentially interstitial carditis. Histopathological analysis of myocardium in KD with repeated endomyocardial biopsy showed interstitial fibrosis, degeneration, disarray and inflammatory cell infiltration [70]. Troponin originates from cardiomyocytes, which explains the elevations of cardiac enzymes such as troponin are rare in acute phase of KD. On the other hand, a high N-terminal portion of B-type natriuretic peptide (NT-pro-BNP) is frequently observed in the acute phase of KD when the inflammatory signs are full-blown. The major stimulus for pro-brain natriuretic peptide (proBNP) secretion in the heart is myocyte stretch in heart failure [71]. Also the brain natriuretic peptide (BNP) is produced in cardiac fibroblasts and increases MMPs [72]. Cardiac fibroblasts play a crucial role in regulating the extracellular matrix of the heart by synthesizing collagen and promoting their degradation by secreting MMP proteins. Ishikawa *et al*. [73] reported that the high level of NT-pro-BNP in acute phase KD is associated with systemic inflammatory response and increased vascular permeability. They also showed that one quarter of KD patients presented with mitral regurgitation (MR) with increasing levels of NT-pro-BNP. As MMP plays an important role in invasion of inflammatory cells by degrading the extracellular matrix, significantly higher levels of MMP-9 was reported in acute phase KD than in sepsis with significantly decreased level through convalescent phase [74].

EMT process is very important in heart development to form mesenchymal cells and to differentiate into fibroblasts, smooth muscle cells and endothelial cells, and EMT process is reactivated in response to the myocardial injury [75, 76, 77, 78]. Epicardium-derived cells are known to be a major source of coronary vascular smooth muscle and cardiac fibroblasts [79]. The endothelium and mesenchyme of atrio-ventricular valves of the heart is made by EMT during the heart development [80, 81]. Valvular heart disease appears distinctively in mitral valve and rarely in aortic valve in 10–25% of the patients with KD which resolves spontaneously [14]. The reason for mitral regurgitation has been inferred from pancarditis with ischemic papillary muscles [5]. Considering lateral leaflet of mitral valve is originated from epicardially derived cells by EMT different from the aortic valve, mitral regurgitation might be more frequently involved in KD.

The most important cardiovascular complication in KD in young children is coronary complications which can be fatal in infants with KD. Prolonged fever during the clinical course of KD may result in coronary arterial dilatation or coronary arterial aneurysms by necrotizing arteritis. This natural history of cardiovascular complications with long-term consequences in KD is well known [14]. In coronary aneurysms in KD, destruction of intima and elastic lamina are involved in aneurysmal formation followed by the subacute or chronic vasculitis, luminal myofibroblastic proliferation or luminal nonocclusive thrombosis that may be complicated with myocardial infarction or complex stenosis. Given that some epicardial cells undergo EMT to form mesenchymal cells to contribute to endothelial and smooth muscle cells of coronary arteries during the development, it is interesting that intima and smooth muscles are involved in coronary arterial lesion in KD. Additionally, as a long-term complication, dilated coronary artery lesions in the acute and subacute phase of KD are more likely to develop subsequent late intima-media thickening due to luminal myofibroblastic proliferation, as seen in intravascular ultrasound and optical coherence tomography, needless to say the cases of giant coronary aneurysms which can be occluded completely [82, 83]. A study has proposed that enhanced EMT and myofibroblast-mediated recruitment of inflammatory cells are involved in the mechanism of coronary artery aneurysm formation mediated by TGF-β [84].

## 4. Conclusions

At first glance, the clinical symptoms of KD may appear to be unrelated phenomena. However, considering that KD belongs to connective tissue disorder uniquely affecting young children whose immune system is not fully matured, and whose dependence on innate immune system is obligatory, heterogenous symptoms and signs presenting only in young patients can be explained. By reviewing the recent research on KD, we presume that the signs of KD present in tissues of non-keratinized stratified squamous epithelium, toward which the sentinel RBCs are recruited as an innate immune response in very young patients (Table 1, Ref. [11, 13, 15, 16, 22, 47, 48, 53, 55, 59, 60, 61, 69, 79, 84]). Also, as oral mucosa and conjunctiva should be healed rapidly with less scar formation under pathogenic environment, premature RBCs containing antifibrogenic factors may be recruited to the non-keratinized mucosa in KD, to regulate the EMT by dermal fibroblasts followed by triggering of the inflammation of connective tissues by circulating RBCs. All of these phenomena seem to be caused by the process of adjusting the balance of the inflammatory response after the stimulus of the yet unknown etiology. Hence, the cardinal symptoms of red lips and conjunctival injection are characteristically present in young children with KD. This may explain why clinical features of KD are rarely present in older age groups whose somatic growth and maturation of individual systems are completed. This review attempts to explain the reason for the clinical features of KD, searching for a link between diagnostic clues of KD with a developmental point of view focusing on the innate immunity with a role of RBCs and EMT in children with KD. Despite extensive research on KD is still ongoing, we hope this perspective provides further clues to solve the puzzle of KD.

**Table 1.** **Major symptoms and signs of Kawasaki disease and tissue borders where epithelial-to-mesenchymal transition is potentially involved**.

Diagnostic guidelines of KD	Involved borders between keratinized and non-keratinized squamous epithelium	Examples of references of organ specific EMT
1. Bilateral bulbar conjunctival injection without exudate	Corneal limbus between Multilayered stratified squamous epithelium of cornea and the non-keratinized stratified epithelium of bulbar conjunctiva	Human limbal mesenchymal cells support the growth of human corneal epithelial stem cells [59]
		Limbus intersects between corneal squamous epithelium and the conjunctival mucous membrane and is purported to harbor corneal stem cells [60]
		Two unique corneal macrophages exhibit distinct characteristics and balance inflammatory responses after epithelial abrasion [61]
2. Red cracking lips, strawberry tongue	Non-keratinized stratified epithelium of vermillion and keratinized perioral skin	Type-2 EMT in oral mucosal inflammatory diseases [15]
		A contrasting role for periostin in wound healing and fibrosis in skin and the oral mucosa [16]
		Faster wound healing and increased extracellular matrix remodeling all contribute to the superior wound healing and reduced scar formation in oral mucosa [47]
		Oral mucosa fibroblasts and dermal fibroblasts had selective differences in cellular behavior and responses to growth factors contributing to the differences in wound healing [48]
3. Polymorphous skin rash or redness on BCG inoculation site	Non-keratinized stratified skin dermis and fibrous scar tissue of BCG	A contrasting role for periostin in would healing and fibrosis in skin and the oral mucosa [16]
		The type-2 EMT in wound healing, tissue regeneration and organ fibrosis as a reparative process in response to ongoing inflammation [53]
		Analysis of factors associated with development of Bacille Calmette-Guérin inoculation site change in patients with KD [55]
4. Red indurative edema of fingers and toes (acute phase), membranous desquamation from fingertips (convalescent phase)	Non-keratinized stratified epithelium under the nails of subungual nail bed and keratinized periungual skin	The type-2 EMT in wound healing, tissue regeneration and organ fibrosis as a reparative process in response to ongoing inflammation [53]
5. Nonpurulent cervical lymphadenopathy	Lymph node capsule and overlying skin and lymphoid follicle	Reprogramming postnatal human epidermal keratinocytes toward functional neural crest fates [13]
6. Interstitial myocarditis, atrioventricular regurgitation, pericardial effusion, coronary artery aneurysms	Interstitium of myocardium, lateral leaflet of atrioventricular valves, pericardium, coronary artery smooth muscle	miRNA was significantly involved in the transforming growth factor-β, epithelial-mesenchymal transition, and cell apoptosis signaling pathways [11]
		Endocardial and epicardial epithelial to mesenchymal transitions in heart development and disease [22]
		The expanding role of the epicardium and epicardial-derived cells in cardiac development and disease [69]
		Epicardium-derived cells contribute a novel population to the myocardial wall and the atrioventricular cushions [79]
		Enhanced EMT and myofibroblast-mediated recruitment of inflammatory cells are involved in the mechanism of coronary artery aneurysm formation mediated by TGF-β [84]

KD, Kawasaki disease; BCG, Bacille Calmette-Guérin; EMT, epithelial-to-mesenchymal transition; TGF-β, transforming growth factor-β.
